# Accurate quantification of 5-Methylcytosine, 5-Hydroxymethylcytosine, 5-Formylcytosine, and 5-Carboxylcytosine in genomic DNA from breast cancer by chemical derivatization coupled with ultra performance liquid chromatography- electrospray quadrupole time of flight mass spectrometry analysis

**DOI:** 10.18632/oncotarget.20093

**Published:** 2017-08-09

**Authors:** Mengzhe Guo, Xiao Li, Liyan Zhang, Dantong Liu, Wencheng Du, Dengyang Yin, Nan Lyu, Guangyu Zhao, Cheng Guo, Daoquan Tang

**Affiliations:** ^1^ Jiangsu Key Laboratory of New Drug Research and Clinical Pharmacy, Xuzhou Medical College, Xuzhou, Jiangsu 221004, China; ^2^ Cancer Institute, The Second Affiliated Hospital, Zhejiang University School of Medicine, Hangzhou 310009, China; ^3^ Jingjiang People’s Hospital, Jingjiang, Jiangsu 214500, China; ^4^ Xuzhou Central Hospital, Xuzhou, Jiangsu 221004, China

**Keywords:** liquid chromatography mass spectrometry, DNA demethylation, breast cancer, derivatization, 5-methylcytosine

## Abstract

The DNA demethylation pathway has been discovered to play a significant role in DNA epigenetics. This pathway removes the methyl group from cytosine, which is involved in the oxidation of 5-methylcytosine to 5-hydroxymethylcytosine (5-hmC) by ten-eleven translocation (TET) proteins. Then, 5-hmC can be iteratively oxidized to generate 5-formylcytosine (5-foC) and 5-carboxylcytosine (5-caC). However, 5-hmC, 5-foC, and 5-caC are hardly detected due to their low content. In this study, we have developed a LC-HRMS method coupled with derivatization to accurately and simultaneously quantify 5-mC levels, along with its oxidation products in genomic DNA. Derivatization was carried out using 4-dimethylamino benzoic anhydride, which has been shown to improve separation and enhance the detection sensitivity. Finally, we successfully applied this method towards the quantification of 5-mC, 5-hmC, 5-foC, and 5-caC in genomic DNA isolated from both human breast cancer tissue and tumor-adjacent normal tissue. We show that 5-foC and 5-caC are increased in tumor tissue. In addition, the levels of 5-mC, 5-hmC, 5-foC, and 5-caC measured in tumor tissue versus tumor-adjacent tissue were found to be distinct among different classifications. This suggests that cytosine modifiers could be used as potential biomarkers for determining the stage of development of breast cancer, as well as prognosis.

## INTRODUCTION

5-Methylcytosine (5-mC) is the predominant DNA modification and is one of the most widely studied epigenetic modifications in higher eukaryotic systems [1]. 5-mC possesses an addition methyl group at the fifth position of the cytosine, which commonly occurs at the CpG dinucleotide site [2, 3]. This modification protrudes into the major groove of the DNA, acting as a potential recognition site for protein binding without altering the Watson-Crick base pairing [4]. In the past decades, 5-mC has been demonstrated to play critical roles in a range of diverse physiological functions [5, 6, 7]. In addition, dysregulation of 5-mC was found in various human diseases, including cancer [8, 9, 10].

5-mC has been well-characterized over the years, and is referred to as the “fifth base” in mammalian genomic DNA. The family of enzymes referred to as DNA methyltransferases (DNMTs) have been shown to be responsible for the generation and maintenance of 5-mC in genomes. These enzymes transfer a methyl group onto the C-5 position of the cytosine [11]. S-adenosylmethionine (SAM) is a common electrophilic methylation donor used in the generation of 5-mC. In addition, 5-mC is a stable and inheritable modification due to its chemical stability. This modification involves the cleavage of a C-C bond that is difficult to occur under mild conditions *in vivo* [12]. However, further research that aimed at understanding DNA demethylation is necessary in order to gain insights into DNA damage repair mechanisms.

In 2009, breakthrough studies first reported the discovery of an active DNA demethylation pathway in mammals [13–15]. This pathway involves the consecutive oxidation of 5-mC by a family of ten-eleven translocation (TET) proteins. In this catalytic reaction, 5-mC is oxidized, resulting in the generation of three intermediate molecules, 5-hydroxymethylcytosine (5-hmC), 5-formylcytosine (5-foC), and 5-carboxylcytosine (5-caC). Both 5-foC and 5-caC can be recognized and further cleaved by thymine DNA glycosylase (TDG) [15–18]. This cleavage event restores the molecule to a normal cytosine through the base-excision repair (BER) process, resulting in the active demethylation of 5-mC [16–19].

As the first oxidation products of 5-mC, 5-hmC has also been demonstrated to play critical roles in cellular differentiation and epigenetic regulation, and its genomic distribution has been shown to be especially enriched in gene bodies and enhancers [20–26]. In addition, 5-hmC levels have been demonstrated in previous studies to be significantly decreased in various tumors [27]. This suggests a critical role of 5-hmC in tumor formation and development. In addition, 5-foC and 5-caC have been shown to have the ability to directly reveal the repair level of C. Some studies have also shown that 5-foC and 5-caC are not only 5-mC oxidation intermediates, but may also act as epigenetic markers [28]. Moreover, the precise functions which 5-hmC, 5-foC, and 5-caC play in evolution and the progression of cancer remain unclear. A quantitative measurement of 5-hmC, 5-foC, and 5-caC in mammals could help to identify related pathogenic mechanisms of tumors [36, 37].

Liquid-chromatography coupled with high resolution mass spectrometry has been frequently used for the quantitative analysis of bio-samples due to the technique’s high selectivity, high resolution, and high mass accuracy. In the past decade, several laboratories have successfully utilized this technique to determine the genome-wide 5-mC in mammals. However, 5-hmC, 5-foC, and 5-caC differ greatly from 5-mC in their abundance, complicating the analysis [29]. According to previous studies, 5-mC accounts for approximately 5% of total 5-C, while 5-hmC is present at a frequency of approximately 10- to 100-fold lower than that of 5-mC. 5-caC and 5-foC levels are even lower, occurring at a frequency of 1 to 20 per 10^6^ cytosine [30, 31]. Thus, quantification of these cytosine modifications are challenging with interference from the highly abundant normal nucleosides. Thus, there is a need for a more sensitive detection method to achieve an accurate quantitative analysis of all cytosine modifications [32].

Chemical derivatization has been widely used in the analysis of LC-MS to not only increase the abundance of samples, but also to enhance sample separation. The chemical derivatization reaction must be convenient, efficient, and be carried out under mild conditions. However, the cytosine is difficult to be derived due to its inherent stable structure. In the field of biochemistry, bisulfite treatment to convert C to T, coupled with sequencing is commonly used to distinguish between 5-C and 5-mC. However, bisulfite treatment is known to be associated with false positives. Thus, researchers have focused on the relative lively side chain of 5-hmC, 5-foC, and 5-caC. For example, Yuan et al. used Girard’s reagents, which easily react with aldehydes and carbonyl groups, to derive the 5-foC and 5-caC. This derivatization has been shown to increase the detection sensitivity of 5-foC and 5-caC in LC-MS analysis. He et al. utilized Friedlander synthesis as a derivatization reaction in order to achieve a bisulfite-free analysis of 5-foC at the genome level [33]. Moreover, few studies have reported on the simultaneous determination of 5-mC, 5-hmC, 5-foC, and 5-caC. One study carried out by Yuan et al. developed an effective method for the chemical derivatization of all cytosine modifications using BDAPE [34], which readily reacts with the 3-N and 4-N positions of cytosine to form a stable penta-cyclic structure. Here, we introduced a classic amidation reaction into the amidogen derivatization of cytosine. Although the amidogen of cytosine has low activity, it has been reported that the anhydride can react with this amidogen with the catalysis of diisopropyl ethylenediamine (DIPEA).

In this work, we have developed a chemical derivation coupled with LC-ESI-HRMS analysis for the sensitive, high resolution, and simultaneous determination of all four cytosine modifications (5-mC, 5-hmC, 5-foC, and 5-caC) in the DNA demethylation pathway. The derivatization was carried out using 4-(dimethylamino) benzoic anhydride, a type of facile and hypotoxicity compound. We optimized the time, temperature, and concentrations used in the reaction to achieve a good derivatization in a short reaction time, with low reaction concentrations, and mild conditions. Our results demonstratedthat the derivatization efficiencies did not change despite the content of the four cytosine modifications. In addition, this quantitative method was developed under the enhancement of the detection sensitivities. The LOD and LOQ of 5-mC, 5-hmC, 5-foC, and 5-caC was found to reach 1.2∼2.5 fmol and 3.7∼7.6 fmol, respectively. Moreover, we used this method for the simultaneous quantification of 5-mC, 5-hmC, 5-foC, and 5-caC in the global human breast cancer and tumor-adjacent normal tissue genomes. We found that the levels of these four cytosine modifications were all increased compared to tumor-adjacent normal tissue, indicating that the increased levels of DNA demethylation could revert the DNA methylation back to the normal degree in breast cancer. In addition, we set the log(m_tumor_/m_adjacency_)as a parameter and discovered an obvious distinction in the different types of cancer under the principal components analysis (PCA). All results from this study demonstrated that 5-mC, 5-hmC, 5-foC, and 5-caC could serve as potential biomarkers for not only for early detection, but also for the classification of breast cancer.

## RESULTS

### Description of the chemical derivatization

**Figure 1:**
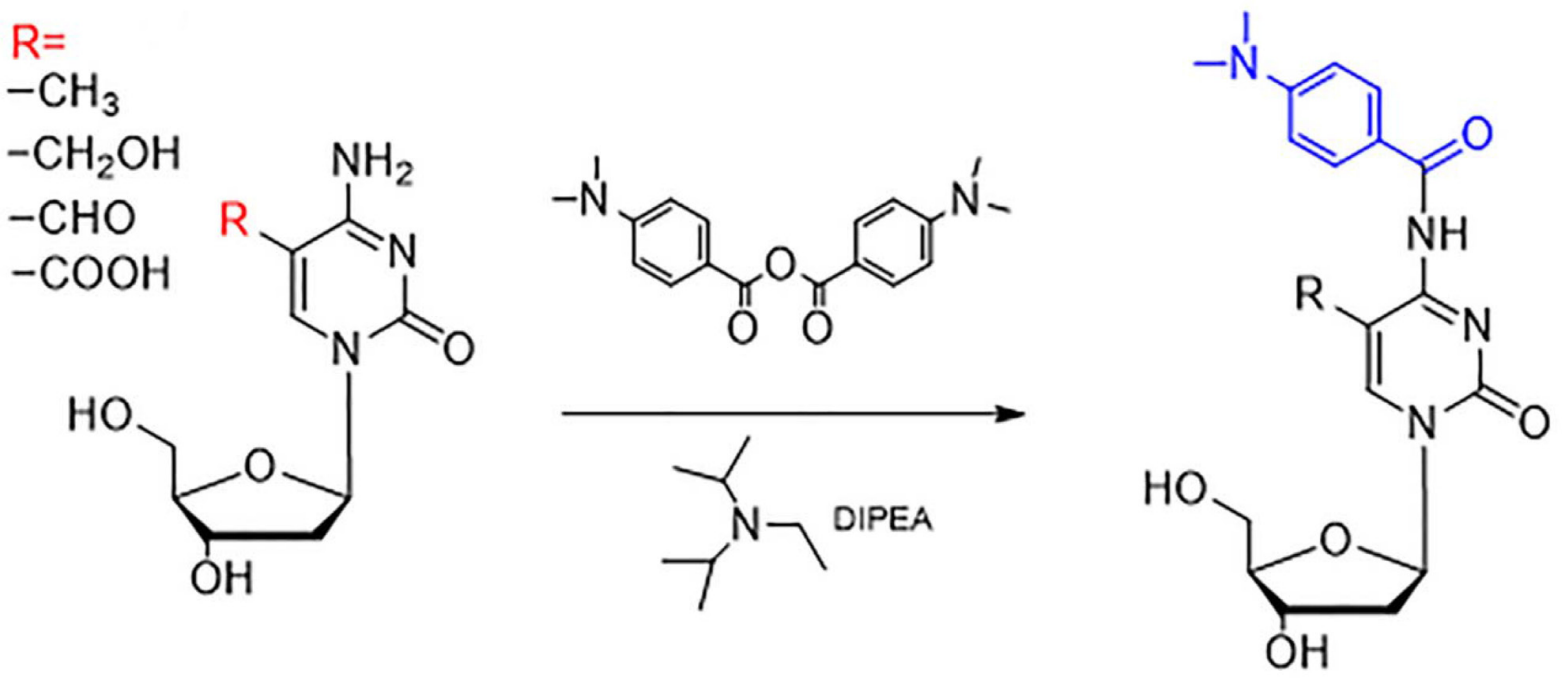
Derivatization of 5-mC, 5-hmC, 5-foC, and 5-caC by 4-dimethylamino benzoic anhydride “R” represents the different groups, including –CH_3_, -CH_2_OH, -CHO, and –COOH.

The modification sites of all four cytosine oxidation products are at the fifth position. The chemical derivatization should locate the subject of cytosine to achieve the simultaneous quantification of these cytosine modifications. Such quantification would facilitate the study of their functions in DNA methylation and the demethylation pathway. In this study, the 4-(dimethylamino) benzoic anhydride was used to react with the amino group of the cytosine. The anhydride group forms an amido bond with the amino group of the cytosine in the fourth position in order to add the 4-(dimethylamino) benzoic group to the cytosine. The entire reaction is carried out under the catalysis of DIPEA (as shown in Figure 1). Four targets were stable in 90°C because the reaction tube was tight and protected by nitrogen. Furthermore, the reaction efficiency was high. Additionally, the introduced 4-(dimethylamino) benzoic group can also result in an increased retention time of the four cytosine oxidation products on RP-LC, improving the separation of these targets. Moreover, the addition of a dimethylamino group can enhance the protonation in mass spectrometry, resulting in increased detection sensitivity.

**Figure 2:**
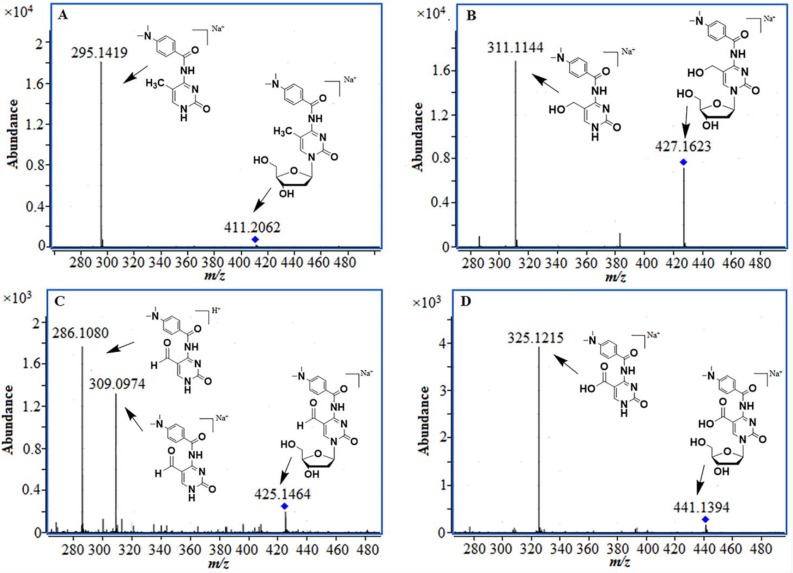
MS/MS results of the derivatization products, (**A**) 5-mC; (**B**) 5-hmC; (**C**) 5-foC; (**D**) 5-caC.

Following derivatizaiton, the four products were then analyzed with ESI-TOF-MS. The accurate mass of 5-mC, 5-hmC, 5-foC, and 5-caC were detected.It was demonstrated that the desired derivatives was expected to be obtained. The derivatization was also verified by the MS/MS results through the fragments (Figure 2, Table 1). These results demonstrated that the error observed between the exact mass and actual mass of the four products was less than 5 ppm in all cases. Besides, the primary fragmentations of them were the loss of ribose at *m/z* 116. All of the results clearly demonstrated that the desired derivatives of 5-mC, 5-hmC, 5-foC, and 5-caC were obtained.

**Table 1:** The accurate mass data of four derivatization product

	Formula	Predicted sodium adduct (*m/z*)	Measured sodium adduct (*m/z*)	Relative Error(ppm)
mC	C_19_H_24_N_4_O_5_	411.1639	411.1648	2.2
hmC	C_19_H_24_N_4_O_6_	427.1588	427.1607	4.5
foC	C_19_H_22_N_4_O_6_	425.1432	425.1440	1.9
caC	C_19_H_22_N_4_O_7_	441.1381	441.1394	3.0

### Optimization of derivatization conditions

Following confirmation of the derivatization reaction, other conditions were optimized to increase the reaction efficiency of the four oxidation products. The conditions optimized included the reaction temperature, the reaction time, and the ratio of the reagent (as shown in Supplementary Figure 2).

In summary, the most ideal simultaneous derivatization conditions for 5-mC, 5-hmC, 5-foC, and 5-caC by 4-(dimethylamino) benzoic anhydride were under 90°C for 3 h with 20 folds of 4-(dimethylamino) benzoic anhydride using DIPEA as the catalyst. Using these optimized derivatization conditions, all four oxidation products of cytosine were found to achieve high derivatization efficiencies (> 95%).

### Optimization of separation and detection conditions under the derivatization

Following the derivatization of targets, we examined and optimized separation and detection of the targets using LC-TOF-MS. From these comparison experiments, we chose the Agilent UPLC column as it exhibited better separation. Acetonitrile solution (A) and water (containing 0.2% formic acid) (B) were selected as the mobile phase components with the gradient elutions: 0–4 min, 15–35% (A); 4–5 min, 35–40% (A); 5–8 min, 45–95% (A). We found that the four targets exhibited shorter separation times under the premise of good retentions, which resulted in a narrower peak width and a higher peak amplitude. 5-mC (4.2 min), 5-hmC (2.6 min), 5-foC (4.0 min), and 5-caC (3.4 min) were all eluted in five minutes, resulting in greatly increased separation efficiency. We also note that 2′-deoxycytidine (dC), 2′-deoxyguanosine (dG) have been identified to also react with 4-(dimethylamino) benzoic anhydride. However, they were not found to affect the separation of the four oxidation products of cytosine. The optimized separation method also demonstrated that these four oxidation products of cytosine have obviously better retention and resolution with derivatization than without it (as shown in Supplementary Figure 1).

Using the optimized separation conditions for these four compounds, the mutual ion suppression from the matrix was minimized, enhancing the detection of the analytes. In addition, the detection is also improved by derivatization. Compared to the native 5-mC, 5-hmC, 5-foC, and 5-caC, derivatization has been shown to increase the ionization efficiencies of these four compounds in ESI by nucleophilic group of 4-(dimethylamino) benzoic anhydride. The limits of detection (LODs) of the derivatives of 5-mC, 5-hmC, 5-foC, and 5-caC were 2.24, 2.53, 2.54, and 1.27 fmol, respectively. The limits of quantitation were 6.70, 7.61, 7.68, 3.68 fmol, respectively. This sensitivity increased the resolution of the mass spectrometry.

### Method Validation

Response linearity of the 4 components was assessed by assaying calibration curves at six concentration levels with five replicates at each level, using enzymolysis followed by the derivatization strategy combined with LC-ESI-HRMS/MS analysis. The results from these experiments demonstrated good linearity within three orders of magnitude, within a range of 1.38∼353 pg/μL for 5-mC, 1.56∼1000 pg/μL for 5-hmC, 1.56∼100 pg/μL for 5-foC, and 0.78∼100 pg/μL for 5-caC. The correlation coefficient (r) was calculated to be greater than 0.99 (Supplementary Table 1).

We also used three different concentrations of QC samples, the low, middle, and high, for accuracy verification. The results showed that both two kinds of QC samples possess good accuracy. The relative error of 5-mC, 5-hmC, 5-foC, and 5-caC were less than 5.65%, 7.20%, 12.0%, and 8.00%, respectively (Supplementary Table 2).

In addition, we used intra- and inter-batch precision to verify the reproducibility of the method. Three different concentrations of QC samples were also treated by the whole strategy and stored for 0 h, 12 h, and 24 h to verify the stability of the samples following derivatization Relative standard deviations (RSDs) were calculated to evaluate the intra- and inter-day precision and the stability of samples. The intra- and inter-batch and stability of the samples RSDs were calculated to be 3.1∼6.3%, 3.4∼5.1% for 5-mC; 0.9∼5.5%, 1.6∼5.5% for 5-hmC; 0.8∼12.9%, 2.3∼9.9% for 5-foC; 5.1∼9.1%, 3.8∼7.2% for 5-caC, respectively. (Supplementary Tables 2, 3)

**Figure 3:**
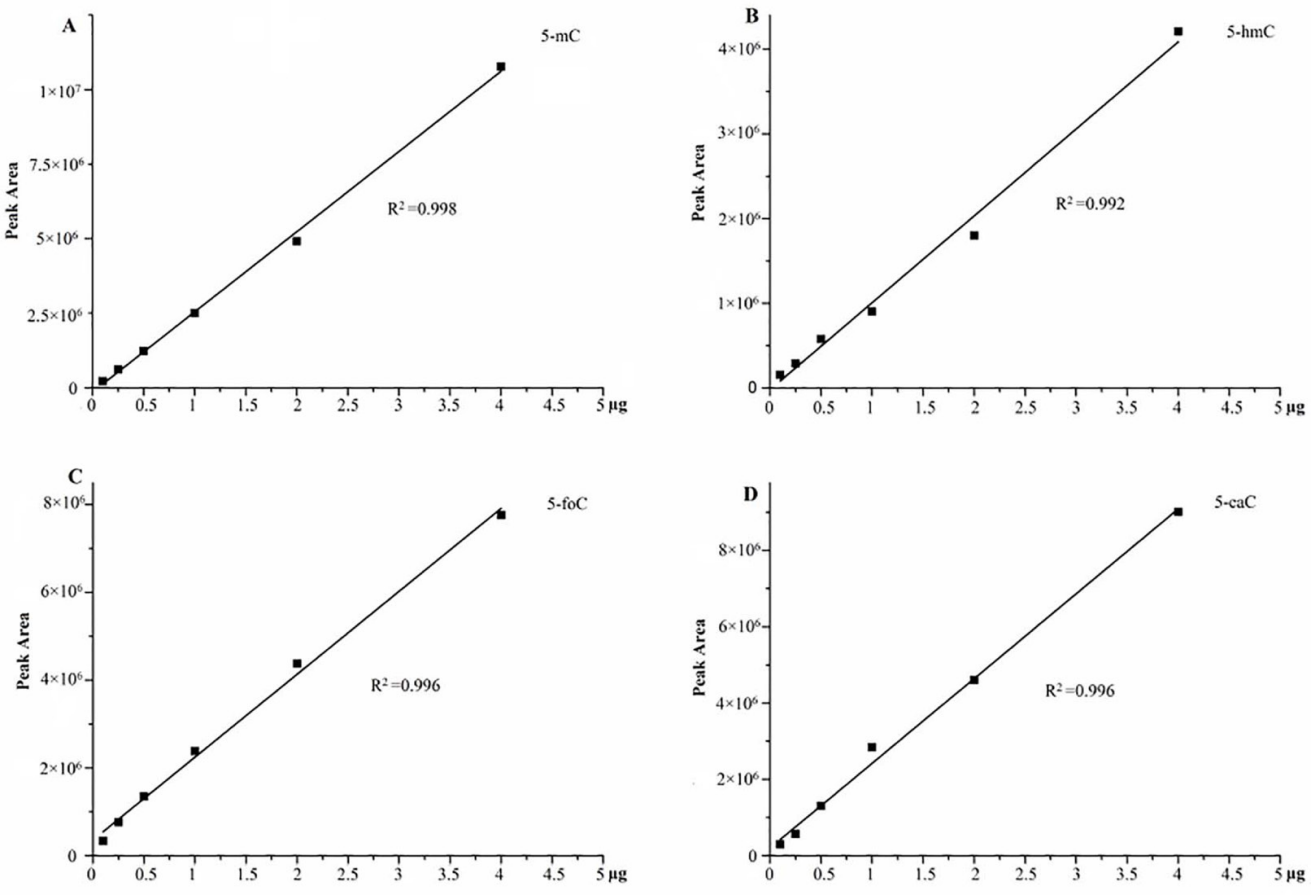
The efficiency of derivatization in different concentrations, (**A**) 5-mC; (**B**) 5-hmC; (**C**) 5-foC; (**D**) 5-caC.

Finally, we designed an efficiency analysis experiment of derivatization in different concentrations to explain the recovery for further verification of our entire analysis strategy. Six different, independent QC sample concentrations were put through the enzymolysis, derivatization, and LC-MS analysis. The results demonstrated that these six samples of each oxidation products of cytosine exhibited excellent linearity, suggesting a good recovery of our entire analysis strategy (as shown in Figure 3).

### Simultaneous quantification of 5-mC, 5-hmC, 5-foC, and 5-caC in genome-wide DNA of human breast cancer and tumor-adjacent tissues

Cytosine methylation and its oxidation products have proved to be important potential biomarkers in cancer, indicating epigenetic alterations. However, there are few studies in existence that have investigated the accuracy of the detection and simultaneous quantification of 5-mC, 5-hmC, 5-foC, and 5-caC in genome-wide DNA studies of human breast cancer tissues.

Herein, we applied our method developed for the simultaneous quantification of 5-mC, 5-hmC, 5-foC, and 5-caC in genome-wide DNA studies of human breast cancer and tumor-adjacent tissues. A total of 26 pairs of freeze-dried breast cancer tissues and matched tumor-adjacent normal tissues were analyzed by DNA extraction, enzymolysis, and derivatization coupled with UPLC-HRMS. In the end, a total of 24 data pairs were effectively detected (Shown in Supplementary Table 4). As the results, the levels of 5-foC and 5-caC were increased in breast cancer tissues (*p* < 0.01), which in keeping with the previous literatures [35].

**Figure 4:**
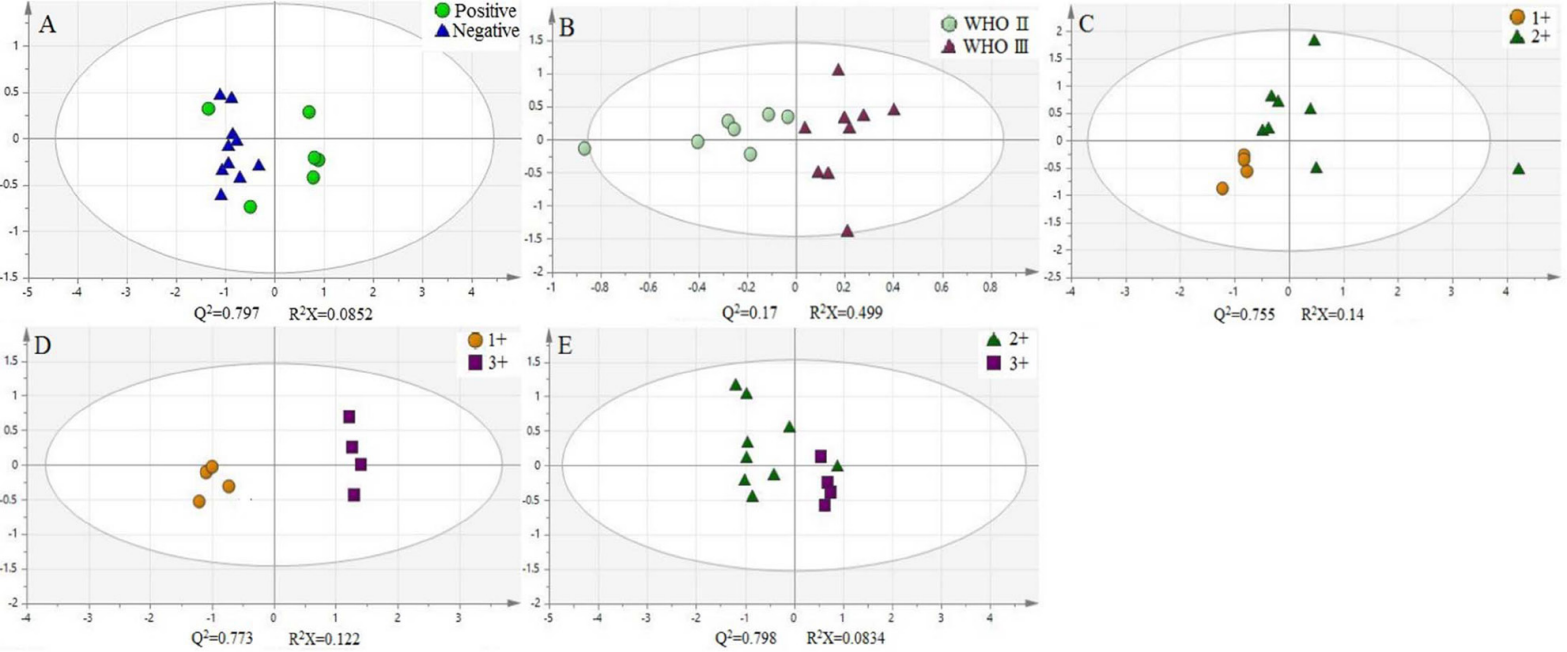
Principal component analysis (PCA) of four oxidation products of cytosine in different classification of human breast cancer, (**A**) Fish type; (**B**) Pathogenic type; (**C**–**E**) C-erbB-2 type.

**Figure 5:**
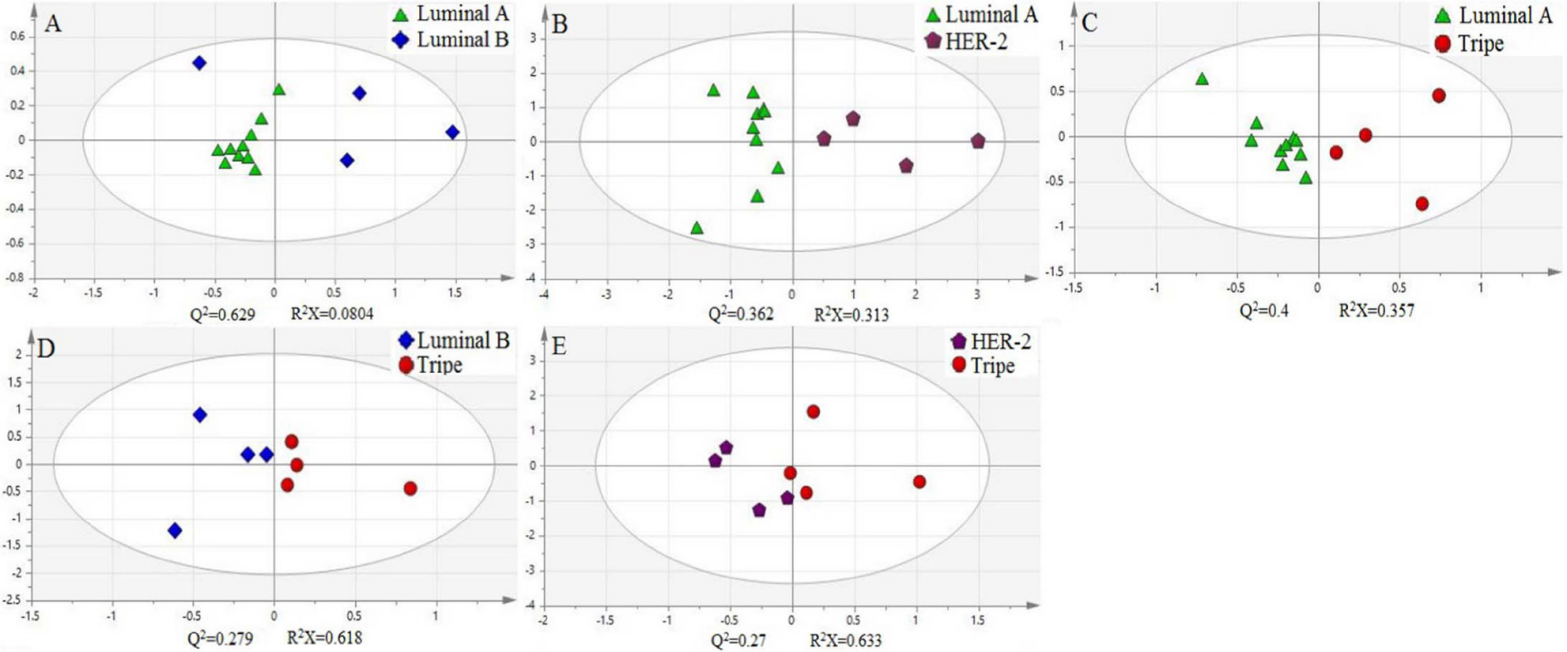
Principal component analysis (PCA) of four oxidation products of cytosine in Molecular subtyping of human breast cancer

In addition, we use principal component analysis (PCA) to further explore these cytosine modifications as potential biomarkers for breast cancer. The experimental data was treated and the log(m_tumor_/m_adjacency_) of 5-mC, 5-hmC, 5-foC, and 5-foC was set as the parameter for each pair of targets. These parameters were grouped according to different classifications. Groups in one classification were put into the analysis at the same time to get the score figure, which reflects the relevance of each group. Interestingly, this treatment of the parameters demonstrated a marked difference between each group from one classification. For instance, the positive group and the negative group in Fish type were distributed in different coordinate quadrants, illustrating the fact that there is a significant distinction between them (Figure 4A). The WHO II and WHO III groups in Pathogenic type could also be distinguished (Figure 4B). If there were more than two groups within one type, the PCA result showed that they also had obvious differences by pair-wise comparison, just like the four groups, Luminal A, Luminal B, Triple-negative, and Her-2 over-expression in Molecular subtyping (Figure 5). Therefore, the quantitative detection of 5-mC, 5-hmC, 5-foC, and 5-caC can not only help to demonstrate the mechanism of breast cancer, but also act as potential biomarkers for the thorough comprehension of breast cancer classification.

Briefly, we have successfully applied our derivatization analysis strategy towards the simultaneous quantification of 5-mC, 5-hmC, 5-foC, and 5-caC in genome-wide DNA studies of human breast cancer and tumor-adjacent tissues. Our results demonstrated that these four cytosine oxidative products possess visible differences between tumor tissues and tumor-adjacent tissues. This makes them promising candidates for potential biomarkers for breast cancer development. Further principal component analysis demonstrated that the content of 5-mC, 5-hmC, 5-foC, and 5-caC from both tumor tissue and tumor-adjacent tissue can be combined to generate an indicator that can be used to distinguish between different cancer classifications.

## DISCUSSION

### Optimization of derivatization conditions

We first optimized the reaction temperature of 5-mC and its three oxidation products. From these experiments, we show that the four modification products all possess different optimal reaction temperatures. In the case of 5-mC, the abundance increased greatly with increased reaction temperatures. However, little change in the abundance was observed with increasing reaction temperatures for 5-hmC. In the case of 5-foC and 5-caC, the variation in abundance was found to be similar, with both exhibiting the highest abundance at a reaction temperature of 70°C. Upon considering the optimal reaction temperature for each, we chose the 90°C as the reaction temperature, especially because 5-mC was found to have the highest abundance at this temperature. In addition, we investigated the optimal reaction time for the derivatization of the four modification products. We showed that the maximum abundance of 5-mC, 5-hmC, 5-foC, and 5-caC was achieved at different reaction times for each. Specifically, 5-hmC, 5-foC, and 5-caC were shown to all have the highest abundance following 3 hours reaction time, while 5-mC achieved its highest abundance following 4 hours reaction time. Considering the efficiency of the entire experiment, we chose 3 hours as the optimized reaction time. Using the optimized reaction temperature and time, we studied the optimal ratio of targets and 4-(dimethylamino) benzoic anhydride. This time, we found that all four targets exhibited the highest abundance when the ratio between them and 4-(dimethylamino) benzoic anhydride was 1:20.

### Optimization of separation and detection conditions under the derivatization

As demonstrated in previous studies, these four oxidation products have relatively poor retention in a C18 reversed-phase chromatographic column. However, the examination results demonstrate that 5-mC, 5-hmC, 5-foC, and 5-caC all possess good retentions in RP-LC following derivatization. We next optimized the specific separation of these products. First, two reverse phase separation columns (Agilent Eclipse Plus C18, 100 × 2.1 mm, 3.5 μm; Agilent UPLC SB C18, 50 × 2.1 mm, 1.8 μm; Agilent Technologies) were compared. Next, different mobile phase components, including methanol/water and acetonitrile/water in combination with formic acid, acetic acid, ammonium formic, and ammonium acetate, were tested. Additionally, different gradient elutions were also tested. The optimization of sample derivatization was also performed using these separation conditions.

Finally, in this work we developed a high resolution and absolute quantification method to simultaneously analyze 5-mC, 5-hmC, 5-foC, and 5-caC by derivatization coupled with LC-ESI-HRMS/MS analysis. For this derivatization, we used an amidation reaction with 4-(dimethylamino) benzoic anhydride to improve the separation achieved with liquid chromatography and to improve the sensitivity detection of mass spectrometry. Following optimization of the derivatization and separation, our method has proven to be accurate, precise, and result in decent recovery. Finally, we used this method for the quantification of 5-mC, 5-hmC, 5-foC, and 5-caC in genomic DNA isolated from human breast cancer tissue and tumor-adjacent normal tissues. The results from these studies demonstrated a significant enhancement of 5-foC and 5-caC in tumor tissue compared to tumor-adjacent normal tissue. This phenomenon could indicate the function of TET protein and DNA demethylation. Thus, we show that these cytosine modifications can be used as potential biomarkers for understanding the mechanism underlying breast cancer. In addition, we identified a relationship between the cytosine modifications and the classification of breast cancer, which should be investigated further in future studies.

## MATERIALS AND METHODS

### Materials

5-hydroxymethyl-2′-deoxy-cytidine (5-hmC), 5-formyl-2′-deoxycytidine (5-foC), and 5-carboxyl-2′-deoxycytidine (5-caC) were purchased from Okeanos Tech Co., Ltd. 5-Methyl-2′-deoxycytidine (5-mC) and 4-dimethylamino benzoic anhydride were purchased from Energy Chemical (Shanghai, China). The oligodeoxynucleotide 5′-ATCGATCG-3′ was purchased from Sangon Biotech (Shanghai) Co., Ltd.

Methanol, acetonitrile (ACN) and Formic acid were of HPLC grade and purchased from MREDA. All other solvents and chemicals used were of analytical grade. N, N-Dimethylformamide (DMF), N, N-diisopropylethylamine (DIPEA) and 4-dimethylaminopyridine (DMAP) were purchased from Energy Chemical (Shanghai, China). Cryonase Cold-active Nuclease and alkaline phosphatase (CIAP) were from Takara Biotechnology Co., Ltd. (Dalian, China). Phosphodiesterase I was purchased from Sigma-Aldrich (Beijing, China). Ultrapure deionized water was purified with Milli-Q water purification system (Millipore, USA).

The blank matrix was prepared by adding H_2_O to oligodeoxynucleotide 5′-ATCGATCG-3′ to give the concentration of 0.2 μg/μL solution. Stock solutions of 5-mC, 5-hmC, 5-foC and 5-caC were prepared in DMF at concentrations of 1mg/mL. Quality control (QC) samples were prepared at concentrations of low, medium and high by spiking the blank matrix solution with the working standard solutions. 4-dimethylamino benzoic anhydride and DMAP were prepared in ACN at concentrations of 1 mg/mL. DIPEA was diluted 10 times by ACN to prepare for use.

### Breast cancer tissues

This study was approved by the Institutional Review Board of Medical Research, The Second Affiliated Hospital, Zhejiang University School of Medicine (SAHZU). All experiments were carried out in accordance with the approved guidelines. A total of 26 pairs of freeze-dried breast cancer tissues and matched tumor-adjacent normal tissues were collected.

### DNA Extraction and Enzymatic Digestion

DNA samples from freeze-dried breast cancer tissues were extracted by Puhe Bio-Tech Co., Ltd. (Wuxi, China) Generally, 10∼30 μg of genomic DNA obtained from each breast cancer tissues. The concentration of the purified genomic DNA was also told along with the samples. DNA solutions were prepared in H_2_O at concentrations of 0.2 μg/μL.

As for enzymatic digestion, genomic DNA (2 μg) was first added 4 units (2 μL) Cryonase Cold-active Nuclease with 10 mM Tris-HCl and 2 mM MgCl_2_, pH 7.5, as the buffer (Cryonase Cold-active Nuclease was diluted 10 times by H_2_O), the mixture was then incubated at 40°C for 1 h. To the resulting solution was subsequently added 1 units (1 μL) of alkaline phosphatase, 0.002 units (1 μL) of phosphodiesterase I (phosphodiesterase I was prepared in alkaline phosphatase buffer at concentrations of 200 mg/mL, alkaline phosphatase buffer was composed of 50 mM Tris-HCl, 10 mM MgCl_2_, pH 9.0), the mixture was diluted to 50 μL by H_2_O. Then, the incubation was continued at 37°C for an additional 4 h. And the enzyme was completely irreversibly inactivated by heating at 65°C for 15 minutes. After adding 2.5 μL of NaCl (3M) and precooled absolute ethanol (125 μL) the obtained solution was placed at −20°C for 30 to 60 minutes. The supernatant was then dried with nitrogen gas at 37°C followed by derivatization. The above operations were in accordance with the enzyme protocol.

### Optimization of Chemical Derivatization

In this study, 4-dimethylamino benzoic anhydride was used to derivatize 5-mC, 5-hmC, 5-foC, and 5-caC. To achieve the best derivatization efficiency, we optimized the derivatization conditions, including reaction temperature and time, and reaction molar ratio between four reactants (5-mC, 5-hmC, 5-foC, and 5-caC) and 4-dimethylamino benzoic anhydride. All the reactions were performed in 100 μL of ACN with 2 μg of four reactants which had been dissolved in DMF (5-mC, 5-hmC, 5-foC, and 5-caC).

As for the effect of reaction temperature on the derivatization efficiency, we investigated a reaction temperature ranging from 30 to 90°C. The derivatization reaction solvent was acetonitrile, its theoretical boiling point was 81–82°C. And the derivatization reaction was a slight reaction, we could only use the micro tube for sealing reaction instead of reflux method. As the atmospheric pressure increases, the boiling point of the solvent increased. However, when the reaction temperature exceeds 90°C, the reaction solvent will still boil. Therefore, 90°C can be set as the highest temperature. The reactions were incubated with 2.5 μL of DIPEA, 6.5 μL of DMAP (1 mg/mL) and 4-dimethylamino benzoic anhydride (molar ratio of 4-dimethylamino benzoic anhydride/ reactants, 20/1) for 3h. The reactions were stopped by immediate adding 20 μL of H_2_O. Then the solution was dried with nitrogen gas and redissolved by 100μL of acetonitrile. Additionally, we optimized the reaction time ranging from 1 to 6 h. The reactions were incubated with 2.5 μL of DIPEA, 6.5 μL of DMAP (1 mg/mL) at 90°C. The reaction molar ratio between four reactants (5-mC, 5-hmC, 5-foC, and 5-caC) and 4-dimethylamino benzoic anhydride was also optimized. We changed the reaction molar ratio between four reactants and 4-dimethylamino benzoic anhydride ranging from 5/1 to 80/1, and the reactions were incubated at 90 °C with 2.5 μL of DIPEA, 6.5 μL of DMAP (1mg/mL) for 3 h.

### The optimization of LC-MS method

LC experiments were performed on Agilent 1290 UPLC (Agilent, America). Two reverse phase separation columns (Agilent Eclipse Plus C18, 100 × 2.1 mm, 3.5 μm; Agilent UPLC SB C18, 50 × 2.1 mm, 1.8 μm; Agilent Technologies) were picked to compare the separation of the derivatization products. The column temperature was set at 30°C. Water containing variable proportion of formic acid and acetic acid (v/v, solvent A) and ACN (solvent B) were investigated to confirm the mobile phase. A gradient of 15–35% ACN for 4 min and 35–40% ACN for 5 min was used. The flow rate of mobile phase was set at 0.3 mL min^-1^. The injection volume was 1 μL.

High resolution mass spectrometric experiments were performed on Agilent 6550 Q-TOF mass spectrometer (Agilent, America). Agilent Data Analysis software version 5.0 was used for the data processing. The detection was performed under positive electrospray ionization (ESI) mode. The nucleosides and derivatives were monitored by MS analysis mode. Solutions were infused from the ESI source at 0.3 mL min^-1^ with parameters: capillary 4000 V, drying gas 12 L min^-1^, drying gas temperature 280°C, Sheath gas temperature 400°C. Nitrogen was used as the nebulizing and drying gas. All MS conditions were optimized to achieve maximal detection sensitivity.

### Quantitative analysis of 5-mC, 5-hmC, 5-foC, and 5-caC in genomic DNA of human breast cancer tissues.

The extracted DNA targets from human breast cancer tissues were performed under the optimized conditions which mentioned above. External standard method was applied referring to previous literatures [34]. We also prepared the quality control (QC) samples to validate the method. All the QC samples were experienced the enzymolysis followed by derivatization using the same procedure as that for genomic DNA of breast cancer. The calibration curve was ploted by the peak area versus amount. The data was also analyzed by *t*-test to confirm the otherness between cancer tissues and cancer-adjacent tissues. Besides, we also treated the data by principal component analysis software (SIMCA 13.0). The number of log(m_tumor_/m_adjacency_) of four cytosine modifications were used to find the distinction between different types of breast cancer.

## SUPPLEMENTARY MATERIALS FIGURES AND TABLES


