# Supplementary material for: Accurate quantification of 5-Methylcytosine, 5-Hydroxymethylcytosine, 5-Formylcytosine, and 5-Carboxylcytosine in genomic DNA from breast cancer by chemical derivatization coupled with ultra performance liquid chromatography- electrospray quadrupole time of flight mass spectrometry analysis
Source: Oncotarget. 2017 Aug 9;8(53):91248–57. doi: 10.18632/oncotarget.20093 (PMC5710920; doi:10.18632/oncotarget.20093)
Supplement: Supplementary file 1 [file oncotarget-08-91248-s001.pdf]

Accurate quantification of 5-Methylcytosine, 5-Hydroxymethylcytosine, 5-Formylcytosine, and 5-Carboxylcytosine in genomic DNA from breast cancer by chemical derivatization coupled with ultra performance liquid chromatography- electrospray quadrupole time of flight mass spectrometry analysis

SUPPLEMENTARY MATERIALS

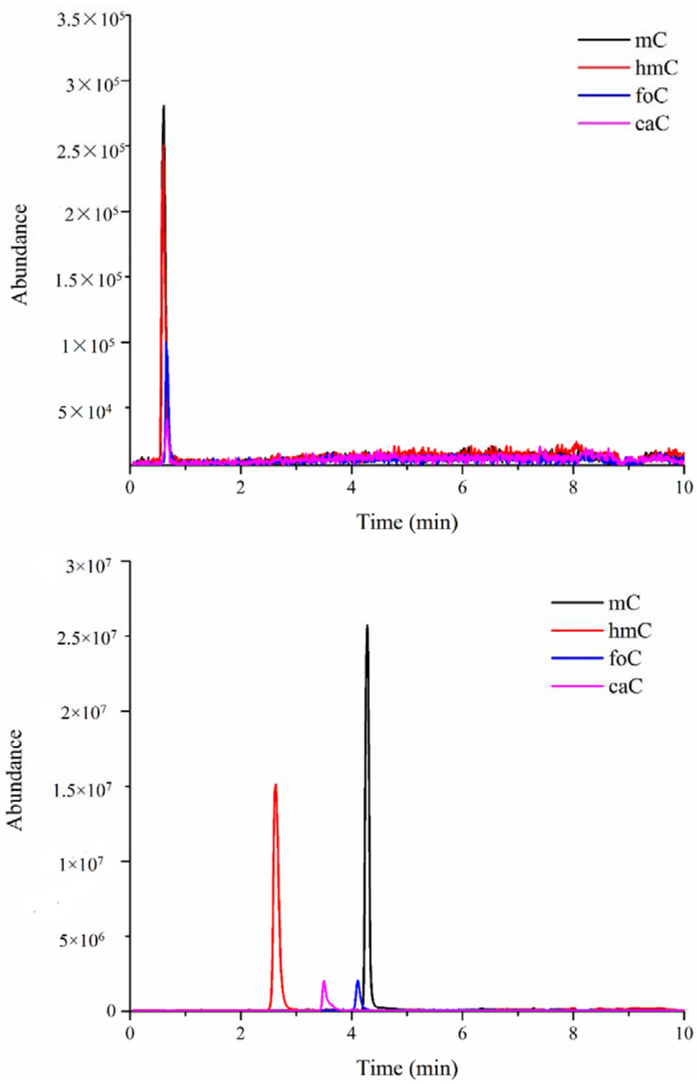

Supplementary Figure 1: LC-MS analysis of 5-mC, 5-hmC, 5-foC, and 5-caC before (**A**) and after (**B**) derivatization under optimized conditions.

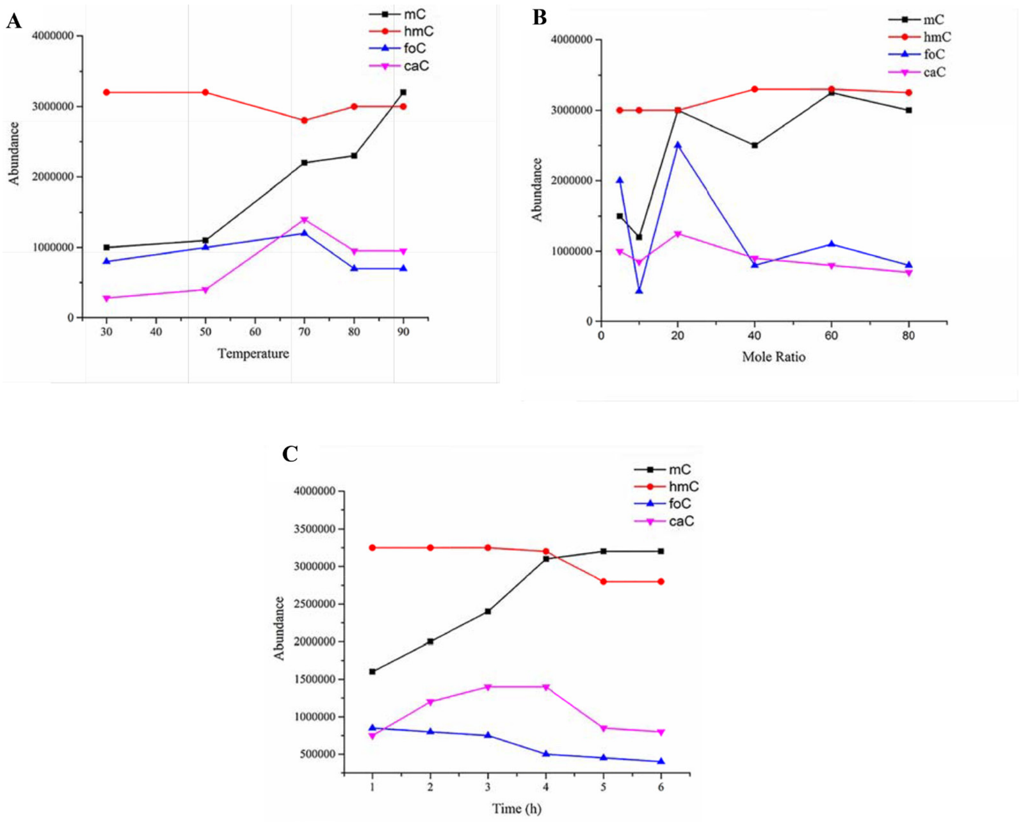

Supplementary Figure 2: Optimization of derivatization conditions for 5-mC, 5-hmC, 5-foC, and 5-caC in (**A**) reaction temperature; (**B**) ratio of the reagent; (**C**) reaction time.

Supplementary Table 1: Linearities of 5-mC, 5-hmC, 5-foC, and 5-caC by derivatization coupled with LC-ESI-MS/MS analysis

Compound	Regression equation	*r*	Linear range (pg/μL)	LLOQ (pg/μL)
mC	y = 478.89x + 3817	0.9986	1.38~353	1.38
hmC	y = 680.73x + 2683.8	0.9997	1.56~1000	1.56
foC	y = 820.66x−599.68	0.9996	1.56~100	1.56
caC	y = 1323x + 2148.4	0.9993	0.78~100	0.78

Supplementary Table 2: Accuracy and stability for the detection of 5-mC, 5-hmC, 5-foC, and 5-foC by derivatization coupled with LC-ESI-MS/MS analysis(pg/μL)

Compound	Theoretical values	24 h		
		Measured values	Accuracy (%)	RSD (%)
	1.5	1.5 ± 0.0	95.96 ± 2.29	2.9
5-mC	22.0	23.0 ± 0.5	104.38 ± 2.74	3.2
	265.5	249.0 ± 5.5	99.34 ± 2.13	2.6
	1.5	1.5 ± 0.0	97.94 ± 2.81	3.5
5-hmC	125.0	135.5 ± 2.0	100.86 ± 1.31	1.6
	750.0	780.0 ± 5.5	106.62 ± 0.76	0.9
	1.5	1.5 ± 0.0	101.11 ± 4.63	5.6
5-foC	12.5	15.0 ± 1.0	100.49 ± 5.34	6.5
	75.0	84.5 ± 1.0	111.78 ± 1.14	1.3
	1.0	1.0 ± 0.0	93.97 ± 4.00	3.8
5-caC	12.5	13.5 ± 0.5	99.98 ± 2.74	3.4
	75.0	74.0 ± 3.5	100.77 ± 4.43	5.4

Supplementary Table 3: Intra- and inter-batch precision for the determination of 5-mC, 5-hmC, 5-foC, and 5-caC by derivatization coupled with LC-ESI-MS/MS analysis (pg/μL)

Compound	Theoretical values	Intra-batch (*n* = 5)			Inter-batch (*n* =15)		
		Measured values	Accuracy (%)	RSD (%)	Theoretical values	Accuracy (%)	RSD (%)
	1.5	1.5	96.650 ± 3.402	6.3	1.5	95.969 ± 2.294	5.1
5-mC	22.0	22.0	98.651 ± 1.137	3.4	22.0	104.389 ± 2.745	4.9
	265.5	250.5	102.458 ± 1.112	3.1	265.5	99.342 ± 2.132	3.4
	1.6	1.6	95.800 ± 4.318	5.5	1.6	97.940 ± 2.811	5.5
5-hmC	125.0	134.0	102.301 ± 1.439	2.1	125.0	100.867 ± 1.319	2.6
	750.0	789.0	104.398 ± 1.336	0.9	750.0	106.626 ± 0.767	1.6
	1.6	1.6	114.794 ± 3.407	12.9	1.6	101.112 ± 4.635	9.9
5-foC	12.5	14.0	99.451 ± 5.372	7.1	12.5	100.495 ± 5.345	5.2
	75.0	79.5	109.378 ± 0.767	0.8	75.0	111.782 ± 1.148	2.3
	1.0	1.0	109.011 ± 15.053	9.1	1.0	93.978 ± 4.009	7.2
5-caC	12.5	13.5	96.988 ± 3.084	6.6	12.5	99.989 ± 2.740	3.8
	75.0	69.5	104.869 ± 2.522	5.1	75.0	100.774 ± 4.430	5.2

Supplementary Table 4: Quantification data of 5-mC, 5-hmC, 5-foC, and 5-caC in genome-wide of 24 pairs of human breast cancer tissues and matched tumor-adjacent normal tissues

NO.	Age	5-mC(ng/2 μg)		5-hmC(ng/2 μg)		5-foC(ng/2 μg)		5-caC(ng/2 μg)	
		tumor	adjacent	tumor	adjacent	tumor	adjacent	tumor	adjacent
1	59	4.947 ± 0.079**	4.532 ± 0.071	1.655 ± 0.085**	1.447 ± 0.122	0.403 ± 0.009**	0.316 ± 0.015	0.062 ± 0.007**	0.030 ± 0.004
2	48	3.190 ± 0.476**	3.177 ± 0.070	1.232 ± 0.114**	1.113 ± 0.008	0.502 ± 0.023**	0.501 ± 0.009	0.179 ± 0.003**	0.176 ± 0.023
3	55	9.983 ± 0.032**	7.512 ± 0.717	4.236 ± 0.276**	3.495 ± 0.266	0.308 ± 0.013**	0.224 ± 0.014	0.030 ± 0.000**	0.016 ± 0.003
4	36	4.955 ± 0.011**	3.984 ± 0.047	2.241 ± 0.108**	1.768 ± 0.020	0.529 ± 0.011**	0.395 ± 0.004	0.142 ± 0.004**	0.087 ± 0.007
5	44	8.879 ± 0.395**	6.605 ± 0.096	1.761 ± 0.089**	1.281 ± 0.077	0.581 ± 0.059**	0.511 ± 0.063	0.221 ± 0.009**	0.178 ± 0.018
6	61	7.601 ± 0.551**	6.206 ± 0.218	1.344 ± 0.160**	1.740 ± 0.098	0.505 ± 0.017**	0.453 ± 0.029	0.261 ± 0.012**	0.226 ± 0.016
7	64	6.804 ± 0.335**	5.553 ± 0.312	4.113 ± 0.265**	3.204 ± 0.032	0.533 ± 0.018**	0.454 ± 0.010	0.149 ± 0.006**	0.087 ± 0.005
8	66	5.228 ± 0.072**	3.661 ± 0.093	2.287 ± 0.175**	1.412 ± 0.120	0.581 ± 0.011**	0.400 ± 0.007	0.145 ± 0.007**	0.030 ± 0.004
9	58	9.301 ± 0.259**	7.101 ± 0.324	4.363 ± 0.472**	2.669 ± 0.167	0.347 ± 0.033**	0.217 ± 0.005	0.023 ± 0.001**	0.004 ± 0.000
10	39	5.080 ± 0.351**	3.699 ± 0.204	2.683 ± 0.018**	1.859 ± 0.023	0.570 ± 0.016**	0.473 ± 0.007	0.158 ± 0.008**	0.085 ± 0.005
11	51	7.219 ± 0.164**	3.696 ± 0.165	1.472 ± 0.228**	1.019 ± 0.103	0.526 ± 0.092**	0.449 ± 0.069	0.241 ± 0.010**	0.087 ± 0.009
12	78	2.600 ± 0.241**	1.176 ± 0.028	0.906 ± 0.086**	0.427 ± 0.065	0.583 ± 0.085**	0.422 ± 0.037	0.248 ± 0.014**	0.190 ± 0.014
13	85	3.188 ± 0.053**	2.077 ± 0.275	0.670 ± 0.026**	0.546 ± 0.065	0.511 ± 0.021**	0.366 ± 0.014	0.197 ± 0.016**	0.109 ± 0.007
14	38	10.745 ± 0.409**	7.427 ± 0.150	3.243 ± 0.224**	2.608 ± 0.139	0.289 ± 0.020**	0.252 ± 0.010	0.021 ± 0.001**	0.002 ± 0.000
15	59	3.427 ± 0.336**	3.248 ± 0.123	2.600 ± 0.064**	1.806 ± 0.116	0.567 ± 0.019**	0.434 ± 0.018	0.043 ± 0.005**	0.012 ± 0.001
16	66	4.984 ± 0.196**	3.899 ± 0.057	3.214 ± 0.181**	2.132 ± 0.102	0.551 ± 0.014**	0.474 ± 0.012	0.172 ± 0.014**	0.023 ± 0.002
17	43	5.350 ± 0.298**	4.658 ± 0.018	3.213 ± 0.241**	2.086 ± 0.143	0.572 ± 0.013**	0.470 ± 0.006	0.125 ± 0.007**	0.059 ± 0.007
18	61	3.081 ± 0.272**	2.582 ± 0.188	0.639 ± 0.111**	0.480 ± 0.036	0.461 ± 0.006**	0.414 ± 0.017	0.096 ± 0.004**	0.085 ± 0.036
19	45	10.231 ± 0.366**	6.023 ± 0.416	4.383 ± 0.321**	2.960 ± 0.204	0.323 ± 0.017**	0.236 ± 0.009	0.021 ± 0.000**	0.005 ± 0.000
20	64	3.813 ± 0.065**	3.516 ± 0.070	2.101 ± 0.105**	1.495 ± 0.088	0.544 ± 0.008**	0.353 ± 0.006	0.122 ± 0.008**	0.029 ± 0.000
21	72	4.023 ± 0.187**	3.345 ± 0.092	0.686 ± 0.002**	0.343 ± 0.057	0.635 ± 0.069**	0.542 ± 0.021	0.199 ± 0.009**	0.128 ± 0.020
22	47	3.436 ± 0.119**	2.481 ± 0.114	0.461 ± 0.029**	0.365 ± 0.062	0.634 ± 0.051**	0.436 ± 0.04	0.225 ± 0.023**	0.166 ± 0.019
23	46	3.969 ± 0.225**	3.490 ± 0.137	0.495 ± 0.065**	0.142 ± 0.007	0.570 ± 0.045**	0.520 ± 0.011	0.229 ± 0.026**	0.184 ± 0.010
24	33	3.415 ± 0.062**	2.392 ± 0.284	0.517 ± 0.031**	0.401 ± 0.065	0.602 ± 0.024**	0.519 ± 0.019	0.232 ± 0.004**	0.176 ± 0.023

**P <0.01 vs.adjacent

Data represent the mean and standard deviation of results from three independent mersurements. **P <0.01 vs.adjacent.
